# Efficacy and safety at 6 months of the XEN implant for the management of open angle glaucoma

**DOI:** 10.1038/s41598-020-61319-1

**Published:** 2020-03-11

**Authors:** Juliette Buffault, Martin Graber, Djawed Bensmail, Élisa Bluwol, Marie-Nathalie Jeanteur, Olivia Abitbol, Nassima Benhatchi, Lauren Sauvan, Yves Lachkar

**Affiliations:** Ophthalmology department, Institut du Glaucome, Groupe Hospitalier Paris-Saint-Joseph, 185 rue Raymond Losserand, 75674 Paris, cedex 14 France

**Keywords:** Glaucoma, Surgery

## Abstract

The purpose of this study is to evaluate the efficacy and complications of the XEN implant as a solo procedure or in association with cataract surgery in patients with open angle glaucoma (OAG). All patients who received a XEN implant between June 2017 and June 2018 were included in the study. The primary and secondary outcomes were: the reduction of the intraocular pressure (IOP) at 6 months postoperatively, the decrease of the glaucoma medications 6 months after surgery, the clinical success rate (eyes (%) achieving ≥20% IOP reduction on the same or fewer medications without secondary surgical intervention), the frequency and type of postoperative interventions as well as the complication rate. We included one hundred and seven eyes from 97 patients with primary OAG (79%), or secondary OAG (21%). Seventy-seven patients (72%) received a standalone XEN implantation and 30 (28%) underwent XEN implantation combined with phacoemusification. The IOP decreased from 20.4 mm Hg ± 6.4 preoperatively to 15.4 mm Hg ± 5.3 six months after the surgery, which represented a reduction of 24.5% (*P* = 1.4.10^−7^). It was associated with a lowering of glaucoma medications from 2.8 ± 1.0 preoperatively to 0.6 ± 1.0 six months postoperatively (*P* = 3.6.10^−34^). The clinical success rate was 67.2% six months after the surgery. The most frequent complications were: IOP spikes >30 mmHg (16.8%), improper position or angled drain (14.0%) and transient minimal hyphema (<1 week) (11.2%). During the follow-up, the needling was required in 34.6% of cases and a total of 10 eyes (9.4%) required a new glaucoma surgery. To conclude XEN implantation appears to be an effective short- and mid-term surgical technique to control IOP in OAG with a low risk of complication. However postoperative maneuvers were frequently required to maintain efficiency.

## Introduction

Glaucoma is the first cause of irreversible blindness^1^. It is a optic neuropathy that affects more than 70 million people world-wide^2^. Nowadays, reducing the intraocular pressure (IOP) is the only effective therapeutic strategy to stop the progression of glaucoma^3–5^, it includes pressure-lowering eye-drops, laser treatments and surgery. In the case of open angle glaucoma (OAG), the most performed glaucoma surgeries are trabeculectomy and non-penetrating deep sclerectomy (NPDS). These two techniques are based on a derivation of the aqueous humor towards the subconjunctival space by creating a filtration bleb (FB). As effective these filtering surgeries are, they are also accompanied by a non-negligible rate of complications such as postoperative bleb leakage, hypotony, and cataract^6–8^. Fibrosis of the bleb is responsible for the majority of surgical failures^7^. Rarely, an infection of the filtering bleb occurs, exposing the eye to a risk of endophthalmitis^7,8^.

Consequently, innovative glaucoma surgery techniques and devices described as “Minimally Invasive Glaucoma Surgeries” (MIGS) have been developed. XEN is one of these new minimally invasive therapeutic option (used for the IOP reduction procedure) created to avoid the pre and post-operative complications and to allow a faster recovery^9,10^. XEN glaucoma stent is a 6 mm long collagen tube with a 45 µm lumen implanted through the anterior chamber into the irido-corneal angle to connect the anterior chamber with the subconjunctival space^11–13^. This new technique avoids complications related to conjunctival dissection, and aims to reduce operating time. Implantation of a XEN is currently indicated as a simple surgery or in combination with phacoemulsification for progressive mild to moderate OAG uncontrolled on topical medications^14^. The XEN received the CE marking in December 2015 and Food and Drug Administration approval in November 2016.

The purpose of this study was to evaluate the efficacy and safety of this new technique within the first 6-month after surgery.

## Patients and methods

This retrospective, non-interventional, monocentric and open-label study was conducted in the ophthalmology department of the Institut du Glaucome in Paris. The research project was submitted to the Ethical Research Medical Group of Paris Saint Joseph Hospital Group who gave its approval on 2018 April, 20^th^ 2018 (local registered number 0310) and ClinicalTrials.gov identifier NCT03733600). All methods were performed in accordance with the relevant guidelines and regulations and a written informed consent from every patient enrolled in the study was obtained.

Patients over 18 with primary open angle glaucoma (POAG), or secondary: pseudo-exfoliative glaucoma (PXG), high myopic glaucoma (HMG) or pigmentary glaucoma (PG) who received a XEN implant between June 2017 and June 2018 as a solo procedure or combined with phacoemulsification were included to the study.

Were excluded patients with angle-closure, congenital and neovascular glaucomas, and aphakia.

The data analyzed comprized preoperative ophthalmic examination including visual acuity, Goldman applanation tonometry, slit lamp examination, gonioscopy, fundus oculi examination, corneal pachymetry, automated visual field (Octopus 900, Haag-Streit Diagnostics, Berne, Switzerland) and RNFL and macular OCT with nodal complex analysis (GCL) (Triton, Topcon Corp., Tokyo Japan). Post-operative evaluations were performed at 1 day, 1 week, 1 and 3 months with visual acuity, Goldman applanation tonometry, slip lamp and posterior pole examination. At 6 months, we performed in addition visual field examination and RNFL, macular GCL OCT. Data concerning intervention and follow-up of patients up to one year after the intervention were recorded.

The primary and secondary outcomes were: the reduction of the intraocular pressure at 6 months postoperatively, the reduction of the glaucoma medications 6 months after surgery, the clinical success rate (eyes (%) achieving ≥20% IOP reduction on the same or fewer medications without secondary surgical intervention) the frequency and type of postoperative interventions as well as the complication rate.

### Surgical technique

Six different surgeons performed the procedures. The standardized surgical technique was described by Buffault *et al*.^15^ as follows: “The procedure was performed under local anesthesia (topical or sub-Tenons). After subconjunctival injection of 0.1 ml mitomycin C (MMC) diluted to 0.1 mg/ml in the superotemporal quadrant, a 2.2 mm inferotemporal corneal incision was made. A miotic (Miostat 100 ®, Carbachol 100 µg, Alcon, Rueil-Malmaison, France) was injected, then the AC filled with viscosurgical device. The preloaded 27-gauge injector was inserted through the incision, then directed to the opposite side of the AC, penetrating the iridocorneal angle, passing through the sclera and arriving in the subconjunctival space approximately 3 mm posterior to the limbus as previous marked, in the target supero-nasal quadrant. The XEN implant was then injected, when the injector retracted. The viscosurgical device (Provisc® OVD, 1% Sodium Hyaluronate, Alcon, Rueil-Malmaison, France) was then removed and the corneal incisions hydrated. At the end of the procedure, 0.1 ml cefuroxime was injected into the anterior chamber. When patients underwent concomitant cataract surgery, the implantation of the stent was performed after placement of the posterior chamber intraocular lens (PCIOL) and injection of the miotic (Miostat 100 ®, Carbachol 100 µg, Alcon, Rueil-Malmaison, France).”

Postoperative treatment included a steroid and antibiotic drop instilled three times a day for 4 weeks (Tobradex®, Dexamethasone 0,1 g and Tobramycine 0,3 g per unit dose, Novartis Pharma, Rueil-Malmaison, France), an anti-inflammatory drop (Indocollyre 0,1% ®, Indometacin, Bausch & Lomb, Montpellier, France) instilled three times a day for 6 weeks and all glaucoma medications were discontinued on the day of surgery. During the follow-up, reintroduction of glaucoma medications or realization of needling was left to the discretion of the surgeon.

### Statistical analysis

We presented results of descriptive analyses as means and standard deviation (SD), median and range for quantitative variables, and as number and percentages for categorical variables. Student’s t test was used to compare means and percentages, and statistical significance was set at *P* < 0.05. We performed statistical analysis through XLSTAT (XLSTAT 2018, Addinsoft. 2018. Paris, France).

## Results

One hundred and seven eyes of 97 patients with POAG (79%), PXG (8%), HMG (3%), PG (2%) or other (7%) underwent a XEN stent implantation. Seventy-seven eyes (73%) benefited from XEN as a solo procedure. Among them, 37 (50.7% of 73) were pseudophakic at the time of surgery. Thirty eyes (27%) benefited from a combined implantation with cataract surgery. Table 1 shows the baseline patient demographic and characteristics.

**Table 1:** Baseline patient demographic and characteristics.

Characteristic		Total (n = 107)
**Demographic**		
	Age mean ± SD (min-max), yrs	68.3 ± 10.8 (36–94)
	Male/Female ratio, (female %) Ethnicity, n (%) − Caucasian − African/Caribbean − Other/mixed	58/49 (45%) 76 (71%) 20 (19%) 11 (10%)
Vision	Preoperative VA (logMAR), mean ± SD	0.7 ± 0.3
**Decision IOP**		
	Mean ± SD, mmHg	20.4 ± 6.1
	Pachymetry mean ± SD, µm	525.7 ± 37.0
Decision medication classes, mean ± SD		2,8 ± 1
**Glaucoma type and severity**		
	Primary open angle	84 (79%)
	Pseudoexfoliative	9 (8%)
	Pigment dispersion	2 (2%)
	High myopia	3 (3%)
	Uveitis	1 (1%)
	Other (basedow, aphakia, steroid induced)	8 (7%)
Cup-to-disc ratio,	Mean ± SD	0.80 ± 0.17
Preoperative MD	Mean ± SD	10.3 ± 6.4
**Previous ocular surgery**		
	Cataract surgery no. (%)	37 (35%)
	Transscleral cyclophotocoagulation no. (%)	1 (1%)
	Trabeculectomy no. (%)	11 (10%)

Preoperative IOP were 20.4 ± 6.4 mmHg with a mean of 2.8 ± 1.0 antiglaucoma medication class.

Eleven patients have had a previous trabeculectomy, one have had a previous diode laser transscleral cyclophotocoagulation. All cataract surgeries were uneventful, and no major complications during implantation of the XEN were experienced.

### Effect on IOP and number of medications

**Figure 1:**
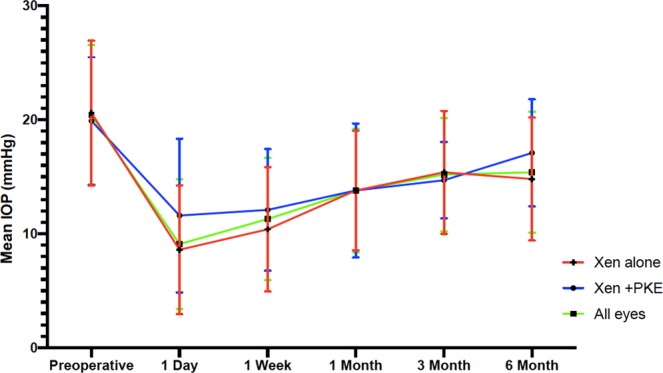
Mean IOP over 6 months of follow-up for all eyes (green, n = 107 eyes), solo-procedure (red, n = 77 eyes,) and combined surgery (XEN + Cataract) (blue, n = 30 eyes). Error bars represent standard deviation in mean IOP. Postoperative IOP values were significantly lower than preoperative values across all time points (t test, P < 0.0001).

**Figure 2:**
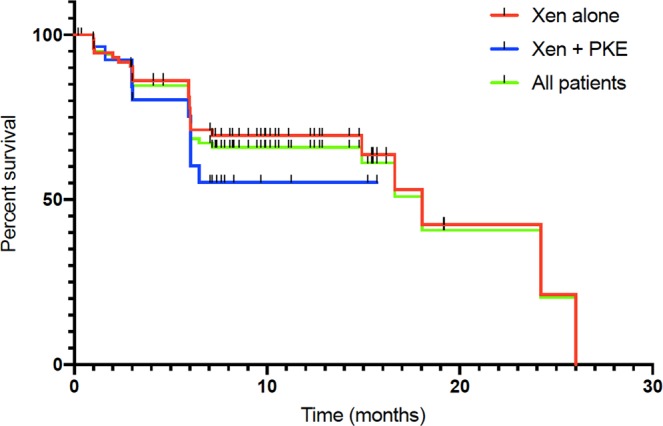
Kaplan-Meier curve showing the probability of achieving success criteria during follow-up for the XEN standalone group (red, n = 77), the combined procedure (blue, n = 30) and all eyes (green, n = 107).

We found a mean 6-month IOP of 15.4 ± 5.3 mmHg. This represented an IOP reduction of 24.4% (*P* = 1,4.10^−7^). Medical treatment was significantly reduced (2.8 ± 1.0 preoperatively, 0.6 ± 1.0 at 6-month (*P* = 3,6.10^−34^). Mean postoperative IOP are presented in Fig. 1, all postoperative IOP values were significantly lower than preoperative values across all time points (t-test, *P* < 0.0001). The clinical success rate defined as eyes achieving ≥20% IOP reduction from baseline on the same or fewer medications without glaucoma-related secondary surgical intervention was 67.2% at 6 months for both techniques, 67.9% for XEN as a stand-alone procedure and 65.5% for the combined procedure (Fig. 2). There was no statistical difference between techniques when it comes to the clinical success rate (Log-Rank, *P* = 0.3).

The IOP lowering was 28% for the 77 eyes that underwent stand-alone procedures, with means of 20.6 ± 6.4 mmHg preoperatively and 14.8 ± 5.4 mmHg at 6 months postoperatively (*P* = 5.3.10^−7^). The average number of glaucoma medications also decreased from 3.0 ± 0.9 prior to implantation to 0.5 ± 0.9 at 6 months (*P* = 4.7.10^−31^). Concerning the 30 eyes that underwent combined XEN and cataract surgeries, the mean IOP reduction wasn’t significant: 14% lowering, with 19.9 ± 5.6 mmHg preoperatively vs. 17.1 ± 4.7 mmHg at 6 months (*P* = 0.087). However, there was a significant decrease in the mean number of antiglaucoma medication from 2.3 ± 0.8 prior to implantation to 0.9 ± 1.2 (*P* = 3.9.10^−6^) at 6 months. The difference in IOP lowering between these two techniques were significant (*P* < 0.05).

Subgroup analysis are presented in Table 2. Neither the Stand-alone versus combined XEN procedure did not show a significant difference in the outcome measures nor did the comparison of patients who benefited from a solo-procedure according to their lens status. However, a statistically lower needling rate can be noted in phakic patients. Analysis ethnicity did not reveal a statistically significant difference in outcome measures.

**Table 2:** Subgroup analysis of XEN efficacy.

	Solo-procedure	Combined procedure	Solo-procedure phakic	Solo-procedure pseudophakic	Caucasian	Non-caucasian
n	77	30	40	37	76	31
IOP preoperatively (mm Hg)	20.6	19.9	20.0	21.2	20.3	20.7
IOP at 6 months (mm Hg)	14.8	17.1	14.2	15.6	15.3	15.7
Number of medications preoperatively	3.0	2.3	2.8	3.2	2.7	2.9
Number of medications at 6 months	0.5	0.9	0.5	0.6	0.7	0.5
Needling (% of patients)	32	40	25*	43*	31	42

^*^Statistically significant difference at p < 0.05.

### Postoperative maneuvers

The frequency and type of postoperative interventions required during the 6-months follow-up period are summarized in Table 3. Needling was performed in 34.6% of cases (37 eyes), 32.5% (25 eyes) after soloprocedure and 40.0% (12 eyes) after combined surgery. Ten eyes (9.4%) required a new filtering surgery or the realization of a cyclo-destructive procedure.

**Table 3:** Postoperative re-interventions and maneuvers.

Postoperative maneuvers	No. (%)
Needling	37 (34.6%)
Improper location requiring re-intervention	4 (3.7%)
Conjunctival suture	1 (0.9%)
Failure requiring repeat surgery	10 (9.4%)

### Complications

The complications encountered are listed in Table 4. The most frequent complications were: transient IOP spike >30 mmHg (16.8%), improper position or angled drain (14.0%), transient minimal hyphema (<1 week) (11.0%), hypothalamia (3.7%), transient hypotony (<1 month) (2.8%), the onset of choroidal detachment or transient choroidal folds (2.8%) and the onset of a cataract (2.8%). Regarding severe complications, only one patient presented a VA loss >2 lines at 6 months (0.9%) it was related with a rise in IOP after surgery which resulted from a bended tube.

**Table 4:** Postoperative complications encountered.

Complications	No. %	
Hypertony > 30 mmHg	18	16.82%
Improper location, angled drain	15	14.02%
Postoperative hyphema	12	11.21%
Flat anterior chamber	4	3.74%
Transient hypotony < 6 mmHg (<1 month)	3	2.80%
Choroidal effusion or folds	3	2.80%
Cataract	3	2.80%
Macular edema	2	1.87%
Chemosis	2	1.87%
Dellen effect	1	0.93%
Implant migration	1	0.93%
Exposure of implant	1	0.93%
Seidel	1	0.93%
Corneal edema > 1 month	1	0.93%
VA loss ≥ 2 lines at 6 months	1	0.93%

## Discussion

We report a mean 6-month IOP of 15.4 ± 5.3 mmHg. This represented an IOP reduction of 24.4%. Medical treatment was significantly reduced (2.8 ± 1 preoperatively, 0.6 ± 1 at 6-month.

In a review of literature regarding the safety and efficacy of the XEN stent^15^ the IOP reduction reported was 25 to 56% depending on the study. Similar results were found in the article of Galal *et al*.^16^: they reported an IOP decrease of 25% at one year after implantation, on 13 eyes with POAG. Pérez-Terregrosa *et al*.^14^ reported an IOP reduction of 29.34% at one year follow-up for 30 eyes of POAG patients undergoing XEN stent implantation combined with phacoemulsification. The modest reduction in IOP in our study can be explain by a lower preoperative IOP comparing to other studies. We also included 10% of patients who had previously had a trabeculectomy failure and 20% of secondary glaucoma who presented a higher risk of post-operative fibrosis.

Interestingly the 6-months IOP reduction was statistically greater in the simple XEN implantation procedures compared to the combined surgeries (28% vs. 14% *P* < 0.05). This difference was also noted by Mansouri *et al*.^17^ who compared the two techniques: 81.0% of the simple XEN implantation and 56.1% of the combined procedures achieved IOP reduction of ≥20% at 1 year (*P* = 0.04). A limitation of our study in the inclusion of both phakic and pseudophakic eye since it could induce methodology bias. Indeed, it is difficult to properly assess the effect of glaucoma surgery on eyes undergoing concomitant cataract extraction, for the mere lens removal is, per se, a short term IOP lowering procedure. A sub-group analysis was therefore performed and no difference was found between phakic and pseudophakic eye who underwent XEN implantation in term of IOP and medication number. Smaller size group in the subgroup analysis may not yield a difference that may exist. However, a statistically lower needling rate could be noted in phakic patients. Widder *et al*.^11^ reported a higher success rate in pseudophakes that underwent XEN implantation (73%) than phakic patients (53%) or those undergoing phacoemulsification combined with XEN implantation (55%).

When comparing the efficacy of this new technique with trabeculectomy and NPDS in the literature, XEN was found to have a more modest IOP lowering effect. Indeed, regarding the efficacy of trabeculectomy on IOP lowering, Kirwan *et al*.^8^ found a IOP a 46% reduction at 2 years, and the TVT study^7^ 48% at one year. Lachkar *et al*.^18^ on 258 eyes operated of NPDS with a follow-up of 6 years found a decrease of 30% IOP at one year, with 47.3% goniopuncture and only 7% needling. However, we cannot conclude that it is less effective since our study did not compare the implant with trabeculectomy or sclerectomy. On their side, Schlenker *et al*.^13^ compared (without randomization) efficacy and complication rates of XEN stent to trabeculectomy on a population with POAG. They reported no significant difference in relative failure risk with XEN implantation compared to trabeculectomy.

In our study, the complications experienced were mild and transient, with no sight threatening complications. Since we do not open the conjunctiva on implantation of a stent, bleb leak is rarer than after trabeculectomy (0.93% in our study vs. 14% and 12% for trabeculectomy in the literature^15^). We also report a low choroidal effusion rate (2,8%). However, the needling rate (34.6%) was higher than those reported in the literature after trabeculectomy, in the study by Kirwan *et al*.^8^ (17% needling) and the TVT study^7^ (14% needling). The comparative study by Schlenker *et al*.^13^ found a higher complications rate in the trabeculectomy group (16%) than in the XEN group (9.7%). They also reported a higher needling rate after a stent (43% vs. 30.8%) compared to trabeculectomy. This greater need of needling can be explained by the absence of conjunctival dissection in the bleb site. In a retrospective study by Gabbay *et al*.^19^, the needling rate after XEN implantation reported 37.7% which is similar to ours but in a 24 months follow-up period. Two elements could explain this. Firstly the 0.02% Mitomycin C used by Gabbay *et al*. was perhaps more effective against fibrosis than the 0.01% that we used. The number of conjunctival erosion reported (4.6%) is also higher in this study which raises the problem of long term complications from the use of Mitomycin in subconjunctival injection without rinsing. Secondly, we have no information on the time between the intervention and the first needling, but in clinical practice, recourse to needling generally takes place in the first post-operative months. It is therefore possible that the 37% of needling reported occurred early during follow-up.

The principal limitation of our study relies on the short follow-up period due to his recent market approval. The IOP at 6 months seems to be a good predictive factor for longer term surgical success of a surgery^20–22^ but we have no long-term efficacy data for the XEN stent in our department. Regarding the complications, the long-term effect of subconjunctival of Mitomycin injection isn’t known, a longer follow up might increase the number of conjunctival erosion and consequently endophthalmitis, as reported by Karri *et al*.^23^. Moreover, endothelial cell density should be monitored given in light of the recent global market withdrawal of the CyPass micro-stent over concerns regarding endothelial cell loss^24^. Another limitation of the study is its retrospective nature which induces a selection bias. The fact that six different surgeons performed the procedure might introduce a lack of standardization in both surgical technique and post-operative care. However, the implantation of a XEN is more standardized than a conventional filtering surgery and the multiplicity of surgeons also better reflects the real life.

Severe cases of glaucoma are often due to late surgeries, and the ability to propose minimally invasive glaucoma surgery (MIGS) for beginner-to-moderate glaucoma combined with phacoemulsification is probably an advance. This faster technique, with an acceptable safety profile and shorter learning time, could be proposed earlier in the management of glaucoma. Unfortunately, the fact that the XEN implantation seems less effective when combined with cataract surgery makes the economic model difficult. To better assess the place of XEN implantation in the management of OAG, other randomized controlled prospective clinical trials are needed.

## Conclusion

XEN implantation appears to be an effective short- and mid-term surgical technique to control IOP in primitive or secondary OAG and to reduce the number of antiglaucoma treatments with an acceptable safety profile. However frequent postoperative maneuvers were required.
